# Comparative Investigation of Anti-Inflammatory Effect of Platelet-Rich Fibrin after Mandibular Wisdom Tooth Surgery: A Randomized Controlled Study

**DOI:** 10.3390/jcm12134250

**Published:** 2023-06-25

**Authors:** Gamze Tanan Karaca, Gonca Duygu, Nilay Er, Eray Ozgun

**Affiliations:** 1 Private Dental Clinic, Canakkale 17110, Türkiye; 2 Oral and Maxillofacial Surgery Department, Faculty of Dentistry, Tekirdag Namik Kemal University, Tekirdag 59030, Türkiye; 3 Oral and Maxillofacial Surgery Department, Faculty of Dentistry, Trakya University, Edirne 22030, Türkiye; 4 Biochemistry Department, Faculty of Medicine, Trakya University, Edirne 22030, Türkiye

**Keywords:** anti-inflammatory effect, edema, serum markers, PRF, wisdom tooth

## Abstract

This study evaluated the anti-inflammatory effect of platelet-rich fibrin (PRF) applied to the extraction socket after impacted mandibular third molar surgery with subjective and objective parameters. Forty-eight patients with impacted wisdom teeth in bilateral and similar positions were included in the study. The control group was formed with the standard surgery and the PRF group was formed with local PRF application in addition to standard procedure (*n* = 96). The anti-inflammatory activity of PRF on postoperative 2nd and 7th days was evaluated subjectively by clinical parameters and objectively by biochemical parameters. Postoperative 2nd- and 7th-day follow-up data of pain, edema, and trismus in the PRF group were found to be statistically significantly lower. Erythrocyte sedimentation rate (ESR) and C-reactive protein (CRP) were found to be statistically significantly lower in the PRF group than the control in the postoperative 2nd-day follow-up period (*p* < 0.001). There was no statistically significant difference in interleukin 6 (IL-6) and tumor necrosis factor-alpha (TNF-α) parameters when the PRF group and the control group were compared in both follow-up periods (*p* > 0.05). The study has demonstrated the effectiveness of locally applied PRF after ITM surgery via clinical parameters and objective data. The quantitative analysis of CRP and ERS can be an effective parameter in determining the amount of inflammation after ITM surgery.

## 1. Introduction

The most frequently performed surgical procedure in oral and maxillofacial surgery is the extraction of the impacted third molars [1]. Minimizing local complications such as pain and swelling after impacted lower third molar surgery has always been the subject of innovative research [2,3,4,5]. The primary cause of these complications is surgical trauma to soft tissue and bone tissue [6]. The causes of such complications are presumed to be due to peri-operative infection, pericoronitis, the difficulty of impaction, duration of the surgery, the technique of removal, perioperative use of antibiotics, etc. [2,7]. Considering the existence of such multiple variables, the necessity of more specific and objective evaluations becomes important. Inflammatory responses-associated surgical trauma can cause biological situations such as increased sedimentation and increased levels of acute phase proteins [8]. The erythrocyte sedimentation rate (ESR) and C-reactive protein (CRP) level, which are among the acute phase reactants, are widely used to show the systemic inflammation status [9,10]. Additionally, cytokines such as interleukin 6 (IL-6) and tumor necrosis factor-alpha (TNF-α) play a crucial role in the acute phase response as they are necessary for initiating the inflammatory response [11]. Serum biomarkers of these cytokines can change quantitatively in the serum during the development of inflammation. Although the increase in the level of these reactants during inflammation is non-specific for diagnostic purposes, it is decisive in monitoring the process and in the follow-up of treatment [12]. 

Recently, autogenous blood concentrations have attracted attention due to their high tissue healing and regenerative effects in medicine and dentistry [13]. Platelet-rich plasma (PRP) is the first generation of platelet concentrates. Although there are lots of benefits of PRP in maxillofacial surgery, its cost and preparation technique are restricting factors for its use routinely [13]. Platelet-rich fibrin (PRF) is known as second generation. Its preparation is simple and inexpensive process [14]. Leukocyte-PRF (L-PRF) is an immune and platelet concentrate that collects all the constituents of a blood sample beneficial for healing and immunity on a single fibrin membrane [15]. L-PRF has many application areas in the branch of dentistry because it has easy clinical usage and it does not require any biochemical treatment [15,16]. Various studies have shown that it contributes to cellular expression with its transforming growth factor-β (TGF- β) and platelet-derived growth factor (PDGF), vascular endothelial growth factor (VEGF), and platelet derived epidermal growth factor (PDGEF) [16,17,18,19,20,21]. In addition to growth factors, several pro- and anti-inflammatory cytokines can also be produced by leukocytes in L-PRF membranes. It has been shown that release of cytokines continue in the three-dimensional architecture of L-PRF, starting from the early inflammatory period up to 21 days. Due to these properties, L-PRF can regulates inflammatory process and increases angiogenesis [22]. In addition, the release of these substances can accelerate tissue healing and reduce the rate of postoperative complications.

Although the use of PRF after the impacted third molar (ITM) surgery is recommended in the literature for benefits such as reducing postoperative swelling and pain, accelerating new bone formation, and soft tissue regeneration, studies on the effect on the severity of inflammation based on objective data have not been fully clarified [23]. In line with this information, this study evaluated the anti-inflammatory activity of PRF in the clinic environment, placed locally in the extraction socket after the bilateral mandibular ITM surgery, on the postoperative 2nd and 7th days. Subjective data were obtained by evaluating clinical parameters such as pain, edema, and trismus; and objective data were obtained by analyzing ESR, CRP, IL-6, and TNF-α serum values. According to our knowledge, this is the first study to explain the anti-inflammatory activity level of PRF by supporting it with objective parameters as well as clinical data. 

## 2. Materials and Methods

This study was approved by Trakya University Clinical Research Ethics Committee with the number 06/09 and was conducted on patients who applied to Trakya University Faculty of Dentistry, Department of Oral and Maxillofacial Surgery between 08.11.2019 and 27.02.2020. All patients included in the study were informed about the purpose and method of the study, and an informed consent form was used to obtain their permission to participate. In the power analysis performed using the G*power 3.1 program, VAS values in the control and PRF groups were found to be between 8% and 25% (alpha error probability = 0.05), and a result of the sample size analysis performed with a power value of 0.8, the total number of samples required to be taken was determined to be 48.

**Figure 1:**
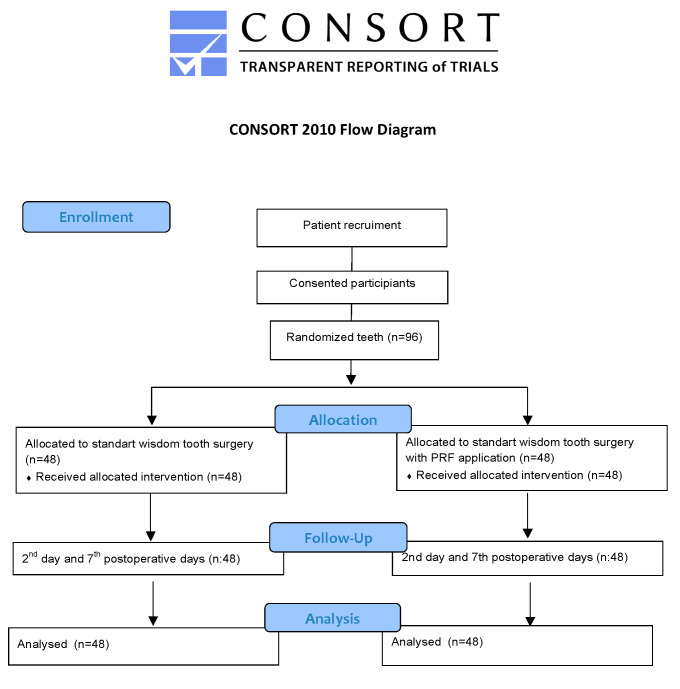
Flow diagram of the study protocol (n: number of impacted tooth surgeries).

The study was completed by extracting a total number of 96 teeth from 48 patients who met all the criteria. Patients between 18 and 50 years old did not have any systemic disease that could affect the healing process, had asymptomatic teeth, and did not smoke with bilateral mandibular ITM in a symmetrical location (vertical and mesioangular position according to Winter classification, and class II, position B and C according to Pell and Gregory classification) (Figure 1) [24] were included in this study. Pregnancy, having a chronic disease, having a local infection in the impacted tooth area, and smoking were exclusion criteria for this study. 

**Figure 2:**
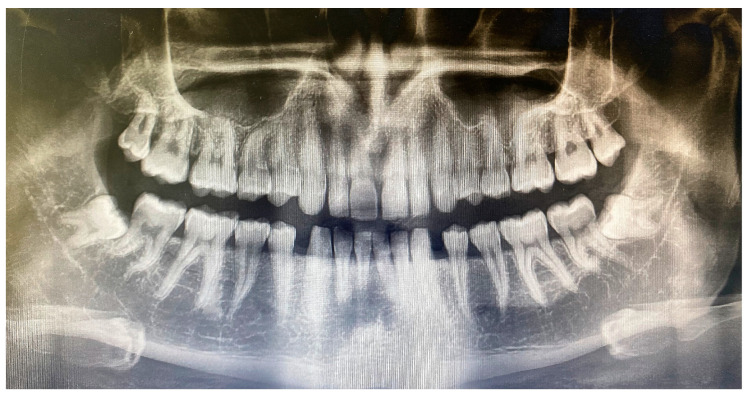
Panoramic radiograph of a patient with a similarly positioned, symmetrical, and bilaterally impacted lower third molar tooth.

The control group was formed by standard extraction of impacted teeth, and the PRF group was formed with local PRF application to the extraction socket in addition to standard impacted tooth surgery (Figure 2). The group the tooth will be included in was determined by the closed envelope method just before the surgery on the first operation day. After 3 weeks, the other impacted tooth of the same patient was extracted with the appropriate surgical procedure and included in the relevant group. During the study, 6 patients were excluded because they did not come to the second appointment, and 4 patients were excluded because the procedure time exceeded 30 min due to root fracture during surgery. In all, 48 patients were included in the study.

### 2.1. Operations

**Figure 3:**
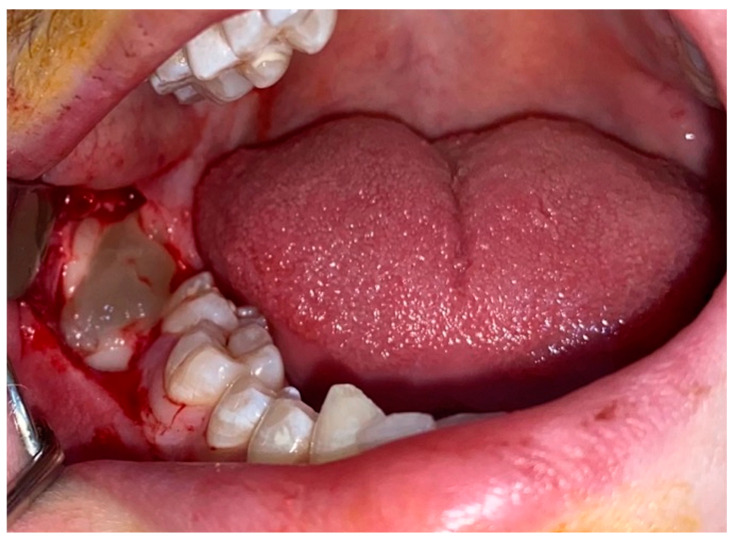
Placement of PRF into the third molar extraction cavity.

All surgical procedures were performed by the same surgeon, with the same flap design and the same surgical technique. Two ml of a local anesthetic solution containing 40 mg/mL articaine HCl and 0.006 mg/mL epinephrine HCl was used for N. alveolaris inferior and N. buccalis blockage. The mucoperiosteal flap was detached by making a horizontal incision starting from the retromolar region, proceeding horizontally in the buccal, circular around the neck of the mandibular second molar, and continuing vertically at the mesial half of the mandibular second molar tooth. Alveolotomy and/or division of teeth and/or roots were performed with sterile tungsten carbide burs with an electric controlled motor rotating at 20,000 rpm under 0.9% saline irrigation during operation. Roots were removed from the alveoli with the help of a bein elevator placed on the buccal and/or mesial parts of the teeth. After tooth extraction, the bone, soft tissue residues, and debris in the area were removed, and the socket was irrigated with 0.9% saline. In the control group, primary suturing was performed after bleeding control without any application to the extraction socket, while in the PRF group, PRF was applied to the socket just before suturing (Figure 3). The position of all impacted teeth and operation duration were also recorded. All patients were prescribed antibiotics (amoxicillin-clavulanic acid, 1 gr, 2 × 1) (Augmentin-BID, GlaxoSmithKline, London, UK), analgesic (Acetaminophen, 500 mg, 3 × 1) (Parol, Atabay, Istanbul, Turkey), and mouthwash (120 mg 0.12% chlorhexidine gluconate and 150 mg 0.15% benzydamine hydrochloride, 200 mL, 3 × 1) (Kloroben, Drogsan, Ankara, Turkey) after the surgical procedure. 

### 2.2. PRF Preparation 

All PRF clots are derived from patients’ own blood sample. Blood sampling was performed through the peripheral antecubital vein by selecting a suitable granule for the patient’s vascular structure with a closed vacuum system. PRFs were prepared according to the method of Choukron et al. [16]. Blood samples (10 mL) were inserted in a centrifuge device (Intra-Lock International Inc., Boca Raton, FL, USA), under 2700 rpm for 12 min using high speed. The platelet-rich fibrin layer remaining between the acellular plasma and red blood cells in the tube was separated with the help of scissors or a scalpel. All PRF were applied clinically similar sized and properly obtained ones were used.

### 2.3. Obtaining Edema, Pain Trismus Levels 

A visual analog scale (VAS) of 100 mm was given to the patients to determine the severity of pain on the operation day and the 2nd and 7th postoperative days, with 0 indicating no pain and 100 indicating the worst pain they had ever experienced. In order to evaluate the severity of edema, the tragus—buccal commissure and lateral canthus—gonion distances of the patients were measured using a flexible ruler before the operation and on the 2nd and 7th days postoperatively, and the results were recorded. To evaluate the trismus level, the interincisal distance of the patients was measured with a flexible ruler before the operation and on the 2nd and 7th days postoperatively in both groups. The progression of swelling and trismus was measured in millimeters and evaluated by comparing it with the value obtained at baseline [25]. 

### 2.4. Obtaining Serum Marker Data

For objective data, 2 mL of the patients’ venous blood was collected before the ITM surgery. ESR values were measured using the Vision ESR analyzer (YHLO Biotech Co., Shenzhen, China), and CRP values were measured using the BN II nephelometric analyzer (Siemens Healthcare Diagnostics, Marburg, Germany). IL-6 levels (pg/mL) were determined using the Human IL-6 Elisa Kit (Elabscience Biotechnology Co., Wuhan, China), and TNF-a levels (pg/mL) were determined using the Human TNF-α Elisa Kit (Elabscience Biotechnology Co., Wuhan, China).

### 2.5. Statistical Evaluation

Data were analyzed with the IBM SPSS^®^ V23 (IBM Company, Chicago, IL, United States) package program. The Mann–Whitney U test was used to compare non-normally distributed data according to paired groups, and an independent two-sample t-test was used to compare normally distributed data. Conformity to the normal distribution was evaluated with the Shapiro–Wilk first test. Spearman’s rho correlation coefficient was used to examine the relationship between non-normally distributed quantitative data. The significance level was taken as *p* < 0.05.

## 3. Results

A total of 48 patients, 31 female and 17 male, with an age range of 19 to 41 (mean age 24.5 ± 4.5 years), underwent mandibular ITM extraction surgery (*n* = 96). There was no statistically significant difference between the PRF group and the control group according to the positions of the impacted teeth and operation durations (*p* > 0.05). The percentages of mesioangular position of ITM were 87.5% in control and 70.8% in PRF group. It was observed that the mean operation time in the control and PRF groups was 17.04 ± 3.18 and 16.98 ± 2.73, respectively.

VAS values in the postoperative 2nd and 7th day follow-up periods in the PRF group were significantly lower than in the control group (*p* < 0.001, *p* = 0.002, respectively) (Table 1).

Edema levels were significantly lower in the PRF group in LC–G measurements in the postoperative 2nd and 7th day follow-up periods (*p* < 0.001, *p* = 0.026, respectively) (Table 2). T-AC measurements in the postoperative 2nd-day follow-up also showed significantly lower results in the PRF group (*p* = 0.021); but in the 7th-day follow-up, the difference was not significant (*p* = 0.179) (Table 2). 

Trismus assessments showed that interincisal distance was significantly higher in the PRF group in the postoperative 2nd and 7th day follow-up periods compared to the control group (*p* < 0.001, *p* < 0.001, respectively), and that PRF had a positive effect in terms of trismus (Table 3). 

The clinical evaluation of inflammatory responses such as pain, edema, and trismus assessment after the ITM surgery is valuable but subjective; these parameters can be affected by many variables. Therefore, in this study, it was aimed to determine the degree of inflammation in the acute phase response of surgical trauma by obtaining objective results based on numerical data as well as clinical measurements. The systemic level of CRP and ESR can be stimulated with surgical trauma and its value can double faster for 8 h and return to normal values within 1 week. Although the increase in the CRP level is faster than the ESR level, the serum levels of both reach their peak on the first or second day. Shortly, it is stated that a moderate inflammation develops after ITM surgery, and it is biochemically effective for up to 1 week [26]. IL-6 and TNF-α are the main cytokines responsible for the production of acute-phase proteins (CRP and ESR) that are commonly expressed following tissue injury in the inflammatory process. For this purpose, serum CRP, ESR, IL-6 and TNF-α were initially measured.

**Figure 4:**
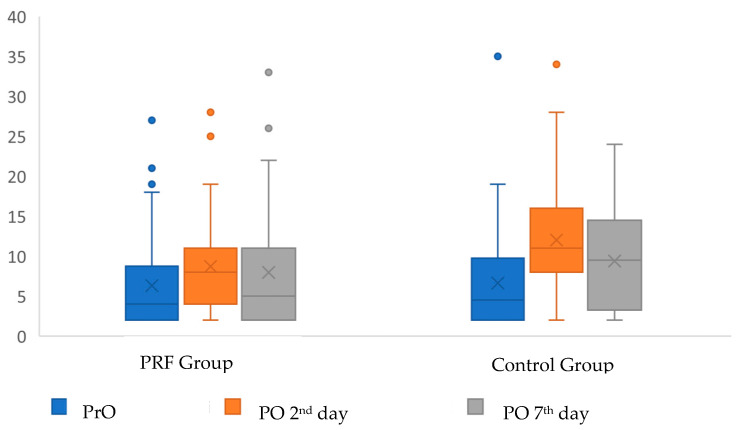
Distribution of ESR values of the groups according to the follow-up periods. PrO: Preoperative, PO: Postoperative. X: mean, Straight line in the box: median, dot: outlier.

**Figure 5:**
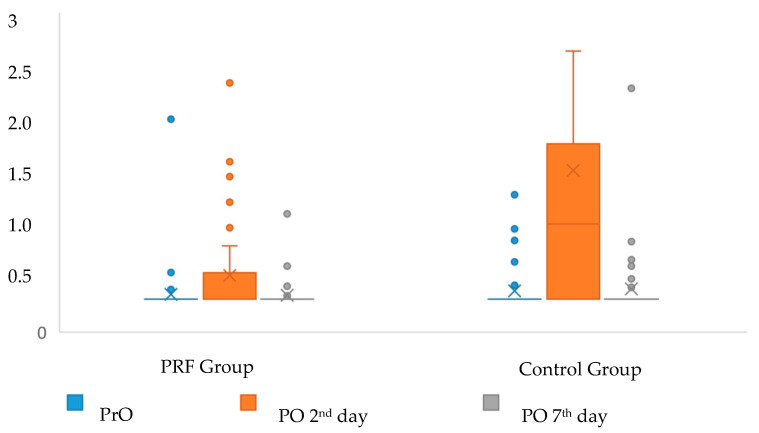
Distribution of CRP values of the groups according to the follow-up periods. PrO: Preoperative; PO: Postoperative. X: mean, Straight line in the box: median, dot: outlier.

Both the increases in serum ESR and CRP values were significantly less in the PRF group on the postoperative 2nd day (*p* = 0.009, *p* < 0.001, respectively), while the increase in the 7th-day levels was not significant in both markers (*p* = 0.158, *p* = 0.345, respectively) (Table 4 and Figure 4 and Figure 5). 

There was no statistically significant difference in IL-6 and TNF-α levels between the two groups in the 2nd- and 7th-day comparisons (*p* = 0.419, *p* = 0.087, *p* = 0.438, *p* = 0.574, respectively) (Table 5). 

Within the PRF group, there was a statistically significant negative moderate relationship between ESR values and trismus assessment and a positive relationship between ESR values and LC–G and T–BC measurements on the 2nd-day follow-up (r = −0.488; *p* < 0.001, r = 0.421; *p* = 0.003, r = 0.523; *p* < 0.001, respectively). There was a statistically significant negatively relationship between ESR values and trismus assessment and a positive relationship between ESR values LC–G and T–BC measurements on the 7th-day follow-up (r = −0.348; *p* = 0.015, r = 0.461; *p* = 0.001, r = 0.485; *p* < 0.001, respectively). Within the control group, there was a statistically significant positive moderate relationship between ESR values and LC–G and T–BC measurements on the 7th-day follow-up (r = 0.384; *p* = 0.007, r = 0.541; *p* < 0.001, respectively). There was no statistically significant difference between other variables (*p* > 0.050) (Table 6).

## 4. Discussion

In the maxillofacial region, ITM surgery is a routine procedure. It may be associated with several postoperative complications, namely, pain, swelling, trismus, alveolar osteitis, or surgical site infection, etc. Many studies have been conducted to identify procedures that can reduce the incidence of these complications. The current study evaluated the anti-inflammatory activity of PRF in clinic environment, which is placed locally in the extraction socket after the bilateral mandibular ITM surgery, on the postoperative 2nd and 7th days. Subjective data were obtained by evaluating clinical parameters such as pain, edema, and trismus, and objective data were obtained by analyzing ESR, CRP, IL-6, and TNF-α serum values. According to our knowledge, this is the first study to explain the anti-inflammatory activity level of PRF by supporting it with objective parameters as well as clinical data.

PRF was first described by Choukroun et al. [15] as an agent that increases wound healing and tissue regeneration. Platelets, growth factors, leukocytes, stem cells, and cytokines in their content support wound healing, angiogenesis, tissue remodeling, bone formation, host defense, and re-epithelialization [27,28,29]. Additionally, PRF has several uses in oral and maxillofacial surgery, since it is obtained from the patient’s blood sample, does not show an allergic or immune response, does not cause cross-reactions, is low in cost and has a short preparation time [30]. These properties of PRF are subjectively supported by many clinical studies. Kim et al. [31] reported that there was no significant difference in reducing the severity of edema and pain when they compared treatment methods with and without PRF application after the bilateral impacted ITM surgery in the same session. In a similar study, Ozgul et al. [32] evaluated the effectiveness of PRF application on the severity of pain and edema after the bilateral ITM surgery and reported that edema in the area where PRF was not applied was higher on the postoperative third day. Jeyaraj et al. [33] also reported that the severity of pain and trismus, as well as edema, were significantly lower in the group that underwent PRF in the postoperative 3rd-day follow-up period after the bilateral impacted ITM surgery. Kumar et al. [34] stated that the edema and pain levels were significantly lower on the PRF applied side after the impacted ITM surgery and PRF increased postoperative comfort. In the current systematic review studies, although there is a general acceptance that the severity of pain and edema was low in the PRF group, the severity of trismus is controversial [26,35]. In our study, the results of VAS and the edema measurement results were significantly lower in the postoperative 2nd and 7th day follow-up periods on the PRF applied side. The high level of pain in the early postoperative period, especially on the 2nd-day follow-up, is consistent with previous studies [32,33,34]. The results of our study on trismus, which gave conflicting results in the literature, again yielded results in favor of the PRF group, and the interincisal distance measurements were significantly higher than those in the control group in the postoperative 2nd and 7th day follow-up periods. 

The clinical evaluation of inflammatory responses such as pain, edema, and trismus assessment after the ITM surgery is valuable but subjective. These parameters can be affected by many variables, including the patient’s cooperation, the investigator’s measurement method, and the appliances required for the measurement, and these factors may affect the results obtained. Therefore, in this study, it was aimed to determine the degree of inflammation in the acute phase response of surgical trauma by obtaining objective results based on numerical data as well as clinical measurements. After third molar surgical intervention, an acute phase and immune response usually develops, which causes an aseptic inflammatory response. Shortly after, it is stated that a moderate inflammation develops after ITM surgery, and it is biochemically effective for up to 1 week [36]. In practice, plasma CRP and ESR levels varies according to the amount and severity of tissue damage, the type of inflammatory stimulus. In healthy individuals, its plasma level is low and rises rapidly on the 2nd day with an acute inflammatory response [37,38]. Although the increase in the CRP level is faster than the ESR level, the serum levels of both reach their peak on the first or second day [39]. Graziani et al., in their study evaluating systemic inflammation biomarkers after ITM surgery, showed that increased white blood cell counts as well as peaks of serum levels of CRP in the first postoperative week [36]. For this purpose, serum CRP and ESR, which are the most frequently used parameters in determining the acute phase response [36], were initially measured. 

In a study evaluating the serum markers and the level of inflammation after surgeries involving osteotomy in the oral cavity, Freitas et al. [37] reported that there was no significant difference in CRP levels at the 48th and 72nd hours after ITM surgery with and without the application of an 830 nm diode laser. Shetty et al. evaluated the CRP level to objectively evaluate the two different surgical techniques outcomes and stated that the quantitative analysis of CRP is an effective parameter in determining amount of inflammation after ITM surgery with the use of piezosurgery in comparison to rotatory osteotomy. They reported that CRP levels in piezo group were higher than preoperative CRP levels but the rise decreased immediately at 24 h. It was stated that the reason for this decrease was due to less surgical trauma in the piezo group [40]. In the current study, the low ESR and CRP values in the PRF group during the 2nd day follow-up period, which is expected to have the highest level of edema, may be explained by the high anti-inflammatory activity of locally applied PRF. The fact that these values were observed at the highest level on the 2nd postoperative day in both groups is consistent with the described highest level of edema time observed after ITM surgery in the literature. Additionally, the absence of a significant difference in these markers on the 7th day supports the idea that they are important parameters only in the early period of inflammatory response. In the PRF group, the ESR value on the 2nd and 7th day was found to be associated with the trismus and edema level, while the CRP value was not found to be associated with clinical parameters. It is normal for the level of ESR, an inflammation marker, to be associated with trismus and edema levels. The observation of this relationship in both groups supports the view that it may be a response to the surgical procedure. However, the lack of a significant relationship between other biochemical markers and clinical parameters can be explained by the low rates of changes in these markers or the late initial follow-up period.

IL-6 and TNF-α are the main cytokines responsible for the production of acute-phase proteins that are commonly expressed following tissue injury in the inflammatory process [41,42]. For this reason, although it is widely known and evaluated in the studies of inflammation research in the medical branch, the number of studies concerning oral surgery is relatively low, and the current information is mostly on oral cancers and tumor development [43]. In a serum marker evaluation study following bone tissue injuries, Karakaya et al. [44] reported that serum CRP and IL-6 levels increased significantly after the first 24 h following lower extremity fractures, the CRP level reached its highest level at the 48th hour, and the IL-6 level returned to normal within 48 h. Dağlı et al. [45] reported that in patients with multiple head trauma, the IL-6 level was highest on the first day, reached the lowest level on the 3rd day, and returned to normal level on the 7th day, while there was no significant change in TNF-α level. These studies are the evaluation of the mentioned serum cytokines in other healing bones. However, healing in the oral environment is different in some respects than in other parts of the body. The number of studies evaluating the local or systemic effects of inflammation on cytokine levels and its relationship with clinical parameters after surgical procedures involving soft and bone tissue in the oral environment is limited. 

Rapid elevation of IL-1α, TNF-α, and IL-6 levels after ITM surgery was demonstrated as the high sensitivity of these markers; thus, this proves that these cytokines are important in the systemic immunological response and can be an important marker in the detection of tissue trauma [8]. It was reported that the amount of local cytokine release affects the level of localized complication in that area and oral surgery procedures can have systemic affects by creating surgical traumatic stress, psychological stress, and anesthetic stress [8]. However, considering this information, the absence of a significant difference in IL-6 levels between the two groups in all follow-up periods in our study can be explained by the fact that IL-6 levels peaked in the first 24 h and returned to normal values after 48 h. Especially in the control group, as the IL-6 level was high in the 2nd-day follow-up period, the IL-6 value could be used as a control in the response of tissue trauma after ITM surgery. In the PRF group, there was no significant difference in IL-6 level at all follow-ups. The fact that no significant difference was observed in the PRF group also supports the view that the tissue damage is less than the control group; that is, PRF has positive effects in limiting local tissue damage. TNF-α levels, on the other hand, did not show a significant difference in postoperative 2nd- and 7th-day follow-ups, similar to previous local tissue trauma results. IL-6 and TNF-α level was not found to be associated with clinical parameters. The absence of any relationship between serum values of cytokines and clinical parameters can be explained by the rapid change of serum values and rapid return of normal values. We think that evaluating these values during earlier follow-ups may be more effective.

This is the first study that has analyzed the quantitative analysis of CRP, ESR, IL-6, and TNF-α in patients undergoing two different surgical techniques (with L-PRF and without L-PRF) to form a basis for scientific quantification. The limitations of the study are the lack of simultaneous surgery and evaluation, which is more suitable for split-mouth studies, and our inability to evaluate the variations that may occur in the patient’s systemic parameters within 3 weeks; however, simultaneous surgery is not possible for such serum marker evaluation. Considering this situation, the number of patients was kept high.

Regardless of these limitations, the study has demonstrated the effectiveness of locally applied PRF after ITM surgery by clinical parameters and objective data. The quantitative analysis of CRP and ERS can be an effective parameter in determining the amount of inflammation after ITM surgery. We think that these objective data could effectively guide clinical studies, especially clinical studies evaluating recovery after ITM surgery with only subjective data.

## 5. Conclusions

In conclusion, local application of PRF after ITM surgery has a significant positive effect in controlling clinical complications, and these results were objectively supported by the measurement of serum ESR, CRP, and IL-6 values in our study. TNF-α levels, on the other hand, did not give a statistically significant difference in terms of the evaluation of clinical parameters.

## Figures and Tables

**Table 1:** Distribution of VAS values of the groups according to the follow-up periods.

	PRF	Control	*p*
PrO	0.0 ± 0.0	0.0 ± 0.0	1.000
	0.0 (0.0–0.0) ^a^	0.0 (0.0–0.0) ^a^	
PO 2nd day	2.88 ± 0.79	4.83 ± 1	<0.001
	3 (1–5) ^b^	5 (2–7) ^c^	
PO 7th day	0.31 ± 0.59	0.71 ± 0.71	0.002
	0 (0–2) ^a^	1 (0–2) ^d^	

PrO: Preoperative; PO: Postoperative; PRF: platelet-rich fibrin; mean ± s. deviation, median, range; different letters in a row indicates significance *p* < 0.05. a–d: There is no difference between groups with the same letter.

**Table 2:** Distribution of LC–G and T–BC values of the groups according to the follow-up period.

	LC–G			T-MC		
	PRF	Control	*p*	PRF	Control	*p*
PrO	11.05 ± 1.01	11 ± 0.98	0.766	15.97 ± 1.49 ^a^	16.03 ± 1.48 ^a^	0.832
	11 (8.8–3.2) ^a^	11 (8.8–13) ^a^		16 (12.4–18.8)	16.05 (12.4–18.8)	
PO 2nd day	11.34 ± 1.04	12.19 ± 1.03	<0.001	16.25 ± 1.53 ^b^	16.98 ± 1.5 ^d^	0.021
	11.4 (9–13.6) ^b^	12 (10–14.8) ^c^		16.4 (13–19)	16.9 (12.6–19.8)	
PO 7th day	11.12 ± 1.03	11.52 ± 0.96	0.026	16.04 ± 1.51 ^c^	16.45 ± 1.46 ^c^	0.179
	11 (9–13.4) ^a^	11.4 (9–13.8) ^d^		16 (12.8–18.8)	16.4 (12.4–19.2)	

PrO: Preoperative, PO: Postoperative; mean ± s. deviation, median, range; different letters in a row indicates significance *p* < 0.05 for each variable. LC–G: Lateral Cantus-Gonion; T–BC: Tragus–Buccal Comissura. a–d: there is no difference between groups with the same letter.

**Table 3:** Distribution of trismus levels of the groups according to the follow-up periods (in millimeters).

	PRF	Control	*p*
PrO	4.73 ± 0.62	4.76 ± 0.63	0.777
	4.65 (3.8–6.2) ^a^	4.8 (3.8–6.3) ^a^	
PO 2nd day	4.09 ± 0.64	3.61 ± 0.69	<0.001
	4.2 (2.2–5.8) ^b^	3.6 (1.8–5) ^c^	
PO 7th day	4.4 ± 0.58	3.86 ± 0.62	<0.001
	4.4 (3.6–5.8) ^d^	3.8 (2.2–5) ^e^	

PrO: Preoperative; PO: Postoperative; different letters in a row indicates significance *p* < 0.05, mean ± s. deviation, median, range. a–d: there is no difference between groups with the same letter.

**Table 4:** Distribution of ESR and CRP values of the groups according to the follow-up periods.

	ESR			CRP		
	PRF	Control	*p*	PRF	Control	*p*
PrO	6.31 ± 5.87	6.63 ± 6.1	0.652	0.35 ± 0.25	0.39 ± 0.21	0.301
	4 (2–7) ^a^	4.5 (2–35) ^a^		0.31 (0.31–2) ^a^	0.31 (0.31–1.29) ^a^	
PO 2nd day	8.71 ± 6.54	12..02 ± 7.36	0.009	0.53 ± 0.45	1.52 ± 1.76	<0.001
	8 (2–28) ^b^	11 (2–34) ^c^		0.31 (0.31–2.34) ^b^	1.02 (0.31–9.44) ^c^	
PO 7th day	7.96 ± 7.09	9.4 ± 6.29	0.158	0.35 ± 0.13	0.41 ± 0.31	0.345
	5 (2–33) ^d^	9.5 (2–24) ^d^		0.31 (0.31–1.11) ^d^	0.31 (0.31–2.29) ^d^	

PrO: Preoperative, PO: Postoperative, mean ± s. deviation, median, different letters in a row indicates significance *p* < 0.05 for each variable. ESR: mm/h, CRP: mg/L. a–d: There is no difference between groups with the same letter.

**Table 5:** Distribution of IL-6 and TNF-α values of the groups according to the follow-up periods.

	IL-6			TNF-α		
	PRF	Control	*p*	PRF	Control	*p*
PrO	10.04 ± 20.09	9.7 ± 19.28	0.702	9.78 ± 6,.4	6.57 ± 3.37	0.094
	6.71 (0.95–31.08)	6.47 (0.17–123.81)		8.9 (3.01–30.23)	5.46 (3.01–13.31)	
PO 2nd day	12.23 ± 28.37	12.27 ± 20.55	0.419	6.13 ± 3.09	7.73 ± 6.65	0.438
	7.11 (0.95–187.7)	7.43 (1.26–135.97)		5.22 (3.01–12.33)	5.71 (3.25–32.19)	
PO 7th day	1.99 ± 25.45	5.52 ± 3.6	0.087	6.81 ± 2.35	8.42 ± 5.1	0.574
	6.5 (1.57–163.58)	4.25 (1.26–15.73)		6.81 (3.25–10.86)	7.18 (3.01–23.86)	

PrO: Preoperative; PO: Postoperative; mean ± s. deviation, median, range; IL-6: pg/mL, TNF- α:pg/m.

**Table 6:** The relationship between VAS, LC–G, and T–BC measurement values, Trismus, and TNF-a, IL−6, CRP, and ESH values in each group and follow-up period.

Group	Follow-Up		VAS		Trismus		LC–G		T–BC	
			r	*p*	r	*p*	r	*p*	r	*p*
PRF group	PrO	ESH	.	.	−0.387	0.007	0.407	0.004	0.447	0.001
		CRP	.	.	0.018	0.903	−0.049	0.739	−0.002	0.990
		IL-6	.	.	0.245	0.127	0.353	0.025	0.245	0.128
		TNF-α	.	.	0.165	0.451	0.059	0.790	0.307	0.154
	PO 2nd day	ESH	0.018	0.903	−0.488	<0.001	0.421	0.003	0.523	<0.001
		CRP	0.054	0.713	−0.240	0.100	−0.237	0.105	−0.261	0.073
		IL-6	−0.123	0.439	−0.020	0.898	0.081	0.610	−0.038	0.812
		TNF-α	−0.098	0.739	0.525	0.054	0.171	0.558	0.303	0.292
	PO 7th day	ESH	0.091	0.538	−0.348	0.015	0.461	0.001	0.485	<0.001
		CRP	−0.073	0.624	0.061	0.682	−0.131	0.374	−0.131	0.376
		IL-6	0.078	0.636	0.060	0.716	0.185	0.259	0.260	0.110
		TNF-α	0.307	0.332	0.340	0.280	0.366	0.243	0.482	0.113
Control group	PrO	ESH	.	.	−0.315	0.029	0.226	0.122	0.331	0.022
		CRP	.	.	0.020	0.891	−0.070	0.638	−0.007	0.962
		IL-6	.	.	0.192	0.242	0.253	0.120	0.191	0.245
		TNF-α	.	.	0.006	0.982	−0.022	0.939	−0.025	0.929
	PO 2nd day	ESH	0.200	0.174	−0.163	0.268	0.280	0.054	0.236	0.106
		CRP	0.034	0.821	0.034	0.816	0.102	0.489	0.117	0.427
		IL-6	−0.212	0.173	−0.157	0.316	−0.183	0.241	−0.189	0.225
		TNF-α	−0.337	0.186	0.020	0.938	0.185	0.476	0.146	0.577
	PO 7th day	ESH	−0.035	0.812	−0.216	0.141	0.384	0.007	0.541	<0.001
		CRP	−0.109	0.461	0.028	0.850	0.045	0.760	0.086	0.563
		IL-6	−0.061	0.703	0.055	0.728	0.238	0.128	0.084	0.598
		TNF-α	−0.134	0.634	0.397	0.142	0.433	0.107	0.448	0.094

PrO: Preoperative; PO: Postoperative; ESR: mm/h; CRP: mg/l; LC–G: Lateral Cantus-Gonion; T–BC: Tragus–Buccal Comissura; IL-6: pg/mL; TNF- α:pg/mL; r: Spearman’s rho correlation coefficient.
